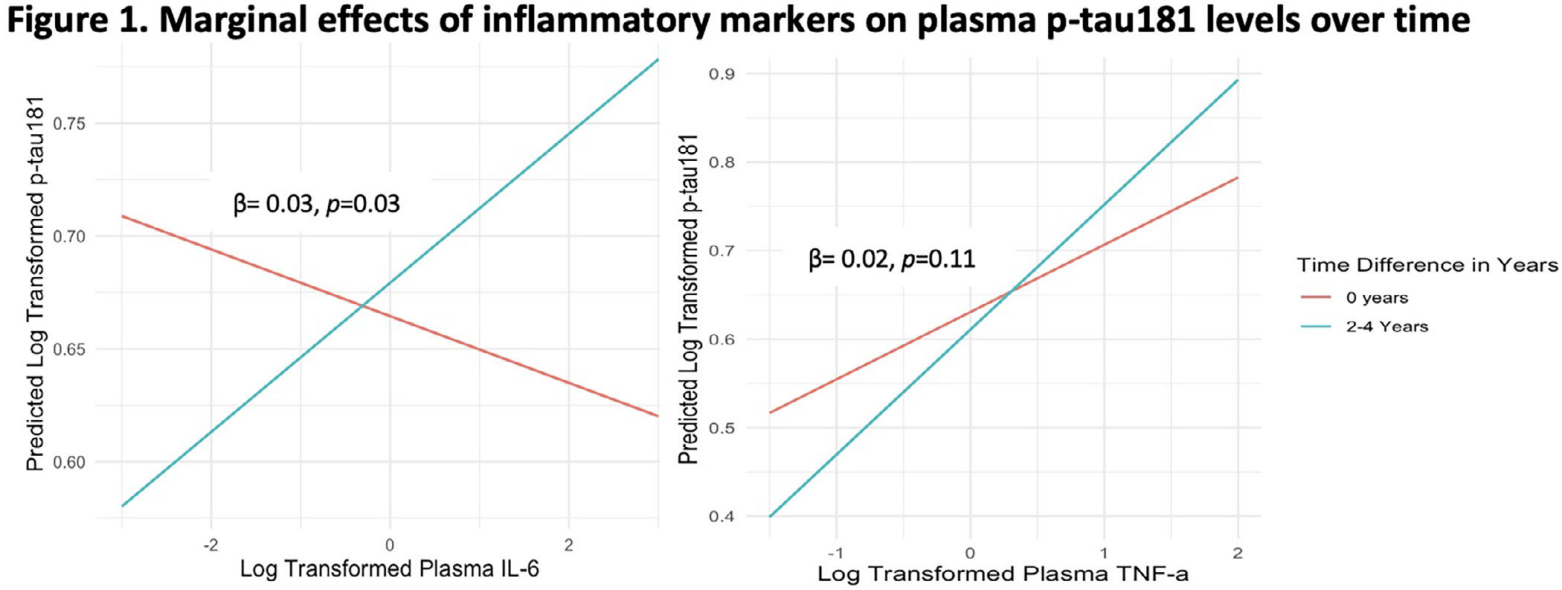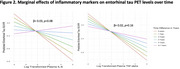# Longitudinal relationships between systemic inflammatory markers, plasma *p* ‐tau181 and entorhinal tau Positron Emission Tomography (PET)

**DOI:** 10.1002/alz70861_108488

**Published:** 2025-12-23

**Authors:** Koral V Wheeler, Danielle Luu, Victoria R Tennant, Noelle Lee, Marylan Davison, Maxwell W Hand, Arthur W. Toga, Sid E. O'Bryant, Kristine Yaffe, Meredith N. Braskie

**Affiliations:** ^1^ Imaging Genetics Center, Mark and Mary Stevens Neuroimaging and Informatics Institute, Keck School of Medicine, University of Southern California, Marina del Rey, CA USA; ^2^ University of North Texas Health Science Center, Fort Worth, TX USA; ^3^ Department of Psychiatry, Neurology, and Epidemiology and Biostatistics University of California San Francisco School of Medicine, San Francisco, CA USA

## Abstract

**Background:**

Plasma levels of tau phosphorylated at threonine 181 (*p* ‐tau181) become elevated before cortical tau and Alzheimer’s Disease (AD) pathology. Associations between systemic inflammation and AD biomarkers are conflicting and suggest protective and disease‐promoting effects. Tumor Necrosis Factor‐alpha (TNF‐a) and Interleukin‐6 (IL‐6) are widely expressed inflammatory markers. Using a longitudinal analysis, we evaluated the separate relationships between plasma TNF‐a and IL‐6 and 1) plasma *p* ‐tau181 and 2) tau positron emission tomography (PET) signal in the entorhinal cortex (an early tau region). We hypothesized that inflammatory markers would have biphasic associations with *p* ‐tau181 and entorhinal tau.

**Method:**

We studied participants at two time points from the Health and Aging Brain Study‐ Health Disparities (HABS‐HD) who were cognitively unimpaired at baseline. Plasma TNF‐a, IL‐6, and *p* ‐tau181 markers were assayed using the Quanterix Single Molecule Array Platform and log transformed. ^18^F‐PI‐2620 tau PET scans were acquired from a Siemens BioGraph Vision scanner and standardized uptake value ratios were calculated in the entorhinal cortex using the inferior cerebellar gray matter as the reference region.

We used linear mixed effects models to investigate the interactions between TNF‐a or IL‐6 and time on plasma *p* ‐tau181 levels (*n* =635, mean age=66, 358 non‐Hispanic White/277 Hispanic), covarying for age, sex, and education. Due to the timing of data collection, participants who had undergone two visits of tau PET acquisition had inflammatory marker data only from their baseline visits. We investigated interactions between either baseline IL‐6 or TNF‐a and time between inflammatory marker and tau PET collections on entorhinal tau (*n* = 296, mean age=70, 33 Black/100 Hispanic/163 non‐Hispanic White), covarying for age, sex, education and Aβ positivity.

**Result:**

Higher IL‐6 was significantly associated with lower baseline *p* ‐tau181 but higher *p* ‐tau181 2‐4 years later (β= 0.03, *p* =0.03) (Figure 1). Higher IL‐6 trended towards associations with lower baseline entorhinal tau PET but higher entorhinal tau 5‐7 years later (β= 0.03, *p* =0.08) (Figure 2). There were no time interactions between TNF‐a and *p* ‐tau181 (β=0.02, *p* =0.11) or entorhinal tau (β=0.02, *p* =0.16).

**Conclusion:**

Among cognitively unimpaired participants, early IL‐6 may be protective in maintaining lower plasma and PET tau levels but sustained inflammation may contribute to later elevated tau.